# Should One Measure Balance or Gait to Best Predict Falls among People with Parkinson Disease?

**DOI:** 10.1155/2012/923493

**Published:** 2012-10-31

**Authors:** Ryan P. Duncan, Gammon M. Earhart

**Affiliations:** ^1^Program in Physical Therapy, Washington University in St. Louis School of Medicine, Campus Box 8502, 4444 Forest Park Boulevard, St. Louis, MO 63108, USA; ^2^Department of Anatomy & Neurobiology, Washington University in St. Louis School of Medicine, St. Louis, MO 63110, USA; ^3^Department of Neurology, Washington University in St. Louis School of Medicine, St. Louis, MO 63110, USA

## Abstract

*Introduction*. We aimed to determine whether gait velocity is as useful as a balance test, a self-report measure of freezing of gait (FOG), and/or a measure of motor symptom severity for predicting falls among people with Parkinson Disease (PD). *Methods*. Fifty-six individuals with idiopathic PD completed a baseline assessment consisting of these measures: (1) MDS-UPDRS III, (2) Mini-BESTest, (3) gait velocity (forward, backward, dual task, and fast), and (4) FOGQ. Retrospective fall history was collected at baseline and six months later. Participants were considered fallers if they had two or more falls in the surveillance period. Ability of the tests to discriminate between fallers and nonfallers was determined using ROC curves followed by pairwise statistical noninferiority comparisons (*P* = .05) of the area under the ROC curve (AUC) for each test. *Results*. At six months, 22% (*n* = 21) of the sample were fallers. Fallers differed significantly from nonfallers on the MDS-UPDRS III, Mini-BESTest, backward gait velocity, and FOGQ. The Mini-BESTest had the highest AUC and was superior to all gait velocity measures at identifying fallers. *Conclusion*. A single measure of gait velocity, even in a challenging condition, may not be as effective as the Mini-BESTest in identifying fallers among people with PD.

## 1. Introduction

 Falls are extremely dangerous and can lead to serious complications for people with Parkinson disease (PD). Complications associated with falls include reduced quality of life, immobility, hip fracture, and mortality [1, 2]. Falling is the most common reason for hospital admission for people with PD [3]. Given the prevalence and negative impact of falls in PD, clinicians must be equipped with measures that are practical for use in clinical settings and accurate in prospectively predicting falls so that interventions can be provided to reduce fall risk.

 A meta-analysis, conducted by Pickering and colleagues, noted that the best predictor of falls in individuals with PD is a history of two or more falls in the previous six months [4]. While it is convenient and informative to gain this information regarding fall history, this is not ideal for the rehabilitation professional because the individual with PD has already fallen. Additionally, simply asking about fall history does not provide the clinician any information regarding factors associated with the cause of the falls. Factors such as postural instability, gait difficulty, and other facets of mobility are significantly associated with falls in people with PD [5, 6]. As such, it is imperative that rehabilitation clinicians employ assessments that test mobility-related constructs in an effort to detect deficits in mobility prior to a fall. Gaining information about future fall risk allows for the implementation of effective rehabilitation programs to reduce fall risk and possibly prevent falls in people with PD.

The ability to predict future falls has improved through use of assessments that include measurements of postural stability during static and dynamic tasks. However, these assessments have some limitations when used in a clinical setting. Balance assessment tools often require special training of the tester who must make subjective ratings of participant's performance. Additionally, administration of balance assessments can be time-consuming [7]. Because of these limitations, there is a need for measures that are objective, quick, and easy to administer. Adopting a measure with these qualities for clinical use must be based on the knowledge that such a measure is equally accurate as more involved measures at predicting falls in people with PD.

 Given the association between impaired gait and falls in people with PD [6], and because it is an objective measure that can be recorded quickly, we asked whether gait velocity is as useful as a multi-item balance test, a self-report measure of freezing of gait (FOG), and/or a measure of motor symptom severity for predicting falls among people with PD. We hypothesized that gait speed would be as accurate as the other measures in prospectively identifying fallers. 

## 2. Methods

### 2.1. Participants

 Participants were recruited from the Washington University School of Medicine Movement Disorders Center and the Volunteers for Health database. Individuals were included if they were diagnosed with “definite” idiopathic PD [8], over the age of 40, and able to walk without an assistive device for 15 meters. Exclusions took place if potential participants reported any of the following: (1) presence of any serious medical condition, (2) musculoskeletal impairment or disease that significantly impaired their ability to walk independently, or (3) history of another neurologic deficit or atypical parkinsonism. Baseline visits for this study were conducted in October-December of 2009, with six-month followups occurring in April-June of 2010. The principal investigator (GME) recruited all participants via phone interview and informed them that they would be participating in a study examining how balance, walking, and other factors influence fall risk in people with PD. Participants were given as much time as they needed to determine whether or not they would agree to participate. All eligible participants provided written and informed consent in accordance with the policies and procedures of the Human Research Protection Office at Washington University.

### 2.2. Study Design

This was a prospective cohort trial. In the Locomotor Control Laboratory at Washington University, participants underwent a baseline assessment of gait and balance conducted by a physical therapist (RPD) trained in the administration of each outcome measure. At baseline, participants were assessed off anti-PD medication, which was considered to be greater than or equal to 12 hours since the last anti-PD medication administration. Six months after baseline, participants reported the number of falls since baseline assessment. 

### 2.3. Outcome Measures

Balance was assessed using the Mini-Balance Evaluation Systems Test (Mini-BESTest), a condensed version of the BESTest and contains 14 items used to measure dynamic balance [9]. Each item is scored on a three-point Likert scale from zero to two with two representing no balance impairment. The Mini-BESTest has high interrater and intrarater reliability when used to assess balance in people with PD [10].

 Walking was evaluated using a GAITRite (CIR Systems, Havertown, PA) walkway in four gait conditions: (1) comfortable forward (FWV), (2) backward (BWV), (3) dual task (DTWV), and fast as possible forward (FastWV). Participants completed three trials for each condition. These three trials were averaged to obtain mean walking velocity for each condition. For DTWV, participants were given a phonemic naming task in which they were asked to name as many words as possible that began with a certain letter. The letter was revealed three seconds before they were instructed to begin walking. The letters used for the three trials were “H,” “L,” and “T.” Walking trials were conducted in the same order for every participant: (1) FWV, (2) BWV, (3) DTWV, and (4) FastWV.

 Motor symptom severity was assessed using the Movement Disorder Society-Unified Parkinson Disease Rating Scale III (MDS-UPDRS III) [11, 12]. The MDS-UPDRS III consists of 33 items, each scored from zero to four, with four representing severe symptoms. From this assessment, each participant was assigned a Hoehn & Yahr (H&Y) stage, with H&Y I indicating minimal PD severity and H&Y V indicating maximal PD severity.

 The Freezing of Gait Questionnaire (FOGQ) is a six-item subjective assessment used to determine presence of symptoms related to freezing of gait and quantify severity of these symptoms. The FOG-Q is a reliable and valid tool that can successfully identify more than 85% of people with PD who experienced FOG [13].

 The physical therapist conducting all assessments recorded fall history via interview. This interview took place only after the participants completed the assessments in order to maintain therapist blinding to fall history. A fall was defined as an unexpected event in which any part of the body contacted the ground. Investigators have previously used this definition in studies of fall prediction in people with and without PD [14, 15]. Each participant chose from the following responses to retrospectively report how many times they had fallen over the six months since baseline assessment: (1) none, (2) one time, (3) 2–10 times, (4) weekly, or (5) daily. Participants were considered fallers if they reported two or more falls. This criterion was chosen because it has been previously used to characterize individuals with PD as fallers [16], and also because any individual might fall once by chance and as such using a single fall to classify people may not be ideal.

### 2.4. Data Analysis

 Independent *t*-tests were used to determine differences between fallers and nonfallers (*P* = .05). Ability of the measures to discriminate between fallers and nonfallers was determined using receiver operating characteristic (ROC) curves followed by pairwise statistical noninferiority comparisons (*P* = .05, one-tailed) of area Under the Curve (AUC) for each test compared to the Mini-BESTest [17, 18]. We chose to use these one-tailed tests because the Mini-BESTest is known to be a good predictor of falls and as such we considered it a reference measure to which the performance of the other measures could be compared. All analyses were conducted using NCSS software (NCSS, LLC, Kaysville, UT, USA).

## 3. Results

 Baseline assessments were completed on 56 participants, of which 12 were fallers. Sample characteristics are presented in Table 1. Fallers and nonfallers differed in H&Y stage, but not age. All individuals who were classified as fallers at baseline were also deemed fallers at six months and all who were nonfallers at baseline were nonfallers at six months. As such, prior history of two or more falls at baseline was a perfect predictor of fall status at six months. 

**Figure 1:**
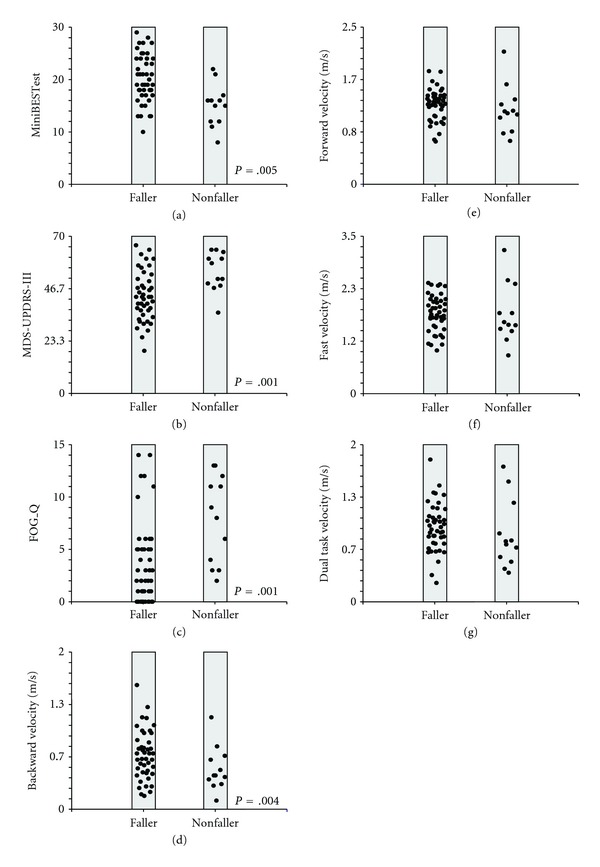
Dot plots for the nonfaller and faller groups for Mini-BESTest (a), MDS-UPDRS-III (b), FOGQ (c), backward walking velocity (d), forward walking velocity (e), fast walking velocity (f), and dual task walking velocity (g). Items in the left column are those that were significantly different between nonfallers and fallers.

At baseline, fallers and nonfallers were significantly different on Mini-BESTest, FOGQ, BWV, and MDS-UPDRS III, but not on FWV, DTWV, or FastWV (Figure 1).

**Figure 2:**
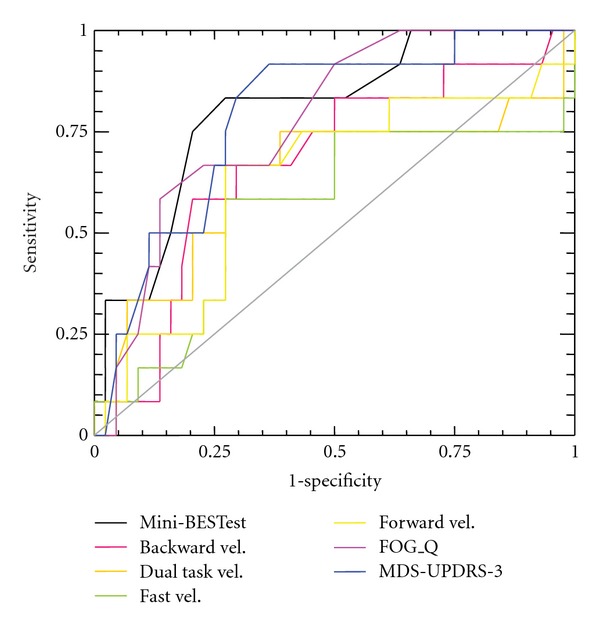
Receiver operator characteristic (ROC) curves for the various measures comparing accuracy in identifying fallers versus nonfallers.

 The Mini-BESTest had the largest AUC, followed by the MDS-UPDRS III, FOGQ, BWV, DTWV, FWV, and FastWV (Figure 2). Noninferiority comparisons of the AUCs showed that the Mini-BESTest was superior to gait velocity in any gait condition but was not superior to FOGQ or MDS-UPDRS III. The MDS-UPDRS III demonstrated the highest positive posttest probability, while FastWV had the lowest (Table 2). The highest negative posttest probability was noted for FastWV; while the Mini-BESTest had the lowest. 

## 4. Discussion

 We aimed to determine if simple measurement of gait velocity, during standard walking or during more challenging gait tasks, would be as accurate as more involved measures in predicting falls among people with PD. Contrary to our hypothesis, gait velocity was not a good predictor of falls. The Mini-BESTest was more accurate than gait velocity, even when measured under challenging walking conditions. While gait impairment has been linked to fall risk in people with PD, our results suggest that gait velocity may not be particularly useful for predicting falls in this group [6]. Perhaps other measures of gait, such as gait coordination or symmetry, would be better at predicting falls in people with mild-to-moderate PD. Asymmetry can, in fact, be present even when gait speed is reasonably intact in people with de novo PD [19]. However, we focused our study on gait velocity because it is a quick, practical measure easily obtained in clinical settings using just a stopwatch. Gait symmetry is more difficult to measure than gait velocity, making it less practical for clinical use. 

It is likely that falls are multifactorial in nature and that sufficient accuracy in predicting falls cannot be obtained through testing only a single construct. Dibble and colleagues reported that a combination of tests, as compared to a single test, resulted in fewer false negatives and greater changes in posttest probability when measuring fall risk in people with PD [20]. This may explain why the Mini-BESTest and MDS-UPDRS III were more accurate than gait velocity in predicting falls. Both the Mini-BESTest and MDS-UPDRS III are batteries of tests that measure PD-specific impairments associated with falling. While it takes more time to complete these types of measures compared to the quick and simple assessment of gait velocity, the limitations of multi-item assessments may be outweighed by the superior predictive information they provide.

In line with previous research noting that a prior history of falls was the best predictor of future falls, our results demonstrated that a history of falls in the past six months was a perfect predictor of future falls [4]. This underscores the importance of inquiring about fall history for all professionals involved in the management of people with PD. However, while the inquiry about fall history is of utmost importance, fall risk assessment should not stop there. The results of this study as well as others demonstrate that the utilization of standardized outcome measures can contribute significantly to the understanding of fall risk for people with PD [20–22]. Additionally, while the results of this study show that these measures are not as accurate in fall prediction as asking about fall history, the Mini-BESTest or MDS-UPDRS III may be employed when the cognitive status of an individual with PD is questionable and self-report of falls may be inaccurate. Finally, information gained through these outcome measures can be used to inform and guide treatment for physical therapists in an effort to reduce fall risk for those with PD.

 This study should be interpreted in the light of the following limitations: (1) sample size was relatively small and consisted of individuals with mild-to-moderate PD, (2) report of fall history relied on retrospective report, and (3) testing only took place when participants off anti-PD medication. Future studies should collect the occurrence of falls on shorter time intervals such as one week or even one day to improve fall report accuracy. Finally, clinicians should consider testing people with PD both on and off medication to gain a full understanding of functional status and vulnerability to falls under both conditions due to known fluctuations associated with anti-PD medications. 

## 5. Conclusion

 A single measurement of gait velocity, in any condition, was less accurate than a more comprehensive balance measure. Future studies could examine whether other aspects of gait (e.g., stride length, symmetry, and interlimb coordination) are more predictive of future falls in people with PD. However, because of the multifactorial nature of falls, a single item may not be as effective as multi-item assessment tools such as the Mini-BESTest. 

## Figures and Tables

**Table 1:** Participant demographics.

	Entire sample (*n* = 56)	Nonfaller (*n* = 44)	Faller (*n* = 12)
H&Y	I (0), II (21), II.5 (25), III (9), IV (1), V (0)	I (0), II (20), II.5 (18), III (6), IV (0), V (0)	I (0), II (1), II.5 (7), III (3), IV (1), V (0)
	Mean: 2.4 (0.5)	Mean: 2.3 (0.05)*	Mean: 2.7 (0.14)*

Age	69.5 (1.2); range 48–89	69.6 (1.2); range 48–89	68.7 (3.1); range 48–82
Gender	32 men/23 women (59% male)	25 men/18 women (59% male)	7 men/5 women (58% male)

Values are mean (standard error) except for H&Y, which follows the format Stage (number of occurrences).

*P < 0.05 (nonfaller significantly different from faller).

**Table 2:** Predictive values and noninferiority comparisons for all outcome measures.

	AUC	Cutoff score	Sensitivity	Specificity	LR+	LR−	Positive posttest probability	Negative posttest probability
Mini-BESTest	0.80	16	0.75	0.79	3.57	0.32	50.18	8.19
MDS-UPDRS III	0.79	58	0.50	0.88	4.17	0.57	54.02	13.81
FOGQ	0.78	8	0.58	0.86	4.14	0.49	53.88	12.10
FWV	0.63	1.17 m/s	0.67	0.72	2.39	0.46	40.29	11.45
BWV	0.68	0.50 m/s	0.67	0.70	2.23	0.47	38.64	11.73
DTWV	0.64	0.78 m/s	0.66	0.72	2.36	0.47	39.93	11.75
FastWV	0.56	1.59 m/s	0.58	0.72	2.07	0.58	36.87	14.13
